# Person Recognition Based on Deep Gait: A Survey

**DOI:** 10.3390/s23104875

**Published:** 2023-05-18

**Authors:** Md. Khaliluzzaman, Ashraf Uddin, Kaushik Deb, Md Junayed Hasan

**Affiliations:** 1 Department of Computer Science and Engineering, Chittagong University of Engineering & Technology, Chattogram 4349, Bangladesh; khalil@iiuc.ac.bd (M.K.); ashrafuddin8778@gmail.com (A.U.); 2 Department of Computer Science and Engineering, International Islamic University Chittagong, Chattogram 4318, Bangladesh; 3 National Subsea Centre, Robert Gordon University, Aberdeen AB10 7AQ, UK

**Keywords:** computer vision, biometrics, gait recognition, deep learning, gait dataset, person recognition, covariate, pattern recognition

## Abstract

Gait recognition, also known as walking pattern recognition, has expressed deep interest in the computer vision and biometrics community due to its potential to identify individuals from a distance. It has attracted increasing attention due to its potential applications and non-invasive nature. Since 2014, deep learning approaches have shown promising results in gait recognition by automatically extracting features. However, recognizing gait accurately is challenging due to the covariate factors, complexity and variability of environments, and human body representations. This paper provides a comprehensive overview of the advancements made in this field along with the challenges and limitations associated with deep learning methods. For that, it initially examines the various gait datasets used in the literature review and analyzes the performance of state-of-the-art techniques. After that, a taxonomy of deep learning methods is presented to characterize and organize the research landscape in this field. Furthermore, the taxonomy highlights the basic limitations of deep learning methods in the context of gait recognition. The paper is concluded by focusing on the present challenges and suggesting several research directions to improve the performance of gait recognition in the future.

## 1. Introduction

Biometrics, a process of identification that relies on unique individual trials, has gained significant attention in recent years due to its significant applications. It basically uses physical or physiological activity to identify individuals and can be categorized into two areas: physical and behavioral [1]. Physical biometrics represents the investigation of physiological traits for identification, whereas behavioral biometrics concentrates on the study of behavioral patterns. Both kinds of biometrics have unique benefits and can be used in conjunction with one another to strengthen security and authentication protocols. Physical biometric methods include approaches such as retina scanning, face recognition [2], fingerprint [3] and iris scanning [4], while behavioral biometric methods include voice recognition [5], keystroke recognition [6], gait recognition [7,8,9,10], and signature recognition [11].

Gait, a behavioral biometric, is a new area of research that looks at how people walk to find out important information about them [1]. Because of this, the process is used for a lot for things such as security, surveillance, law enforcement, health, sports, and identifying people [12].

The different sensing modalities, such as wearable sensors, are used to obtain the gait data from the video sequences [13]. The non-wearable system basically uses an imaging sensor to capture gait information [14]. The process is called vision-based gait recognition. In this paper, we have focused on the present state of the art of published literature that is based on vision-based gait recognition, and the backbone of its functionality lies in deep learning techniques. This review paper aims to provide a comprehensive overview solely of the current state of the field of gait recognition based on vision-based gait recognition, with a focus on deep architectures and their limitations. To date, different literature reviews have mainly focused on the two methodologies for gait recognition: model-based and model-free processes, a model-based strategy, and a holistic approach without the use of models. In order to make a model-based method robust to noise and occlusion, it is built on extracting dynamic information about the human anatomy from the images and tracking changes in these structures over time, while a holistic approach considers the complete human body’s motion pattern. It is computationally efficient and can manage low-resolution images, making it better suited for outdoor surveillance than model-based approaches.

There are several problems with gait recognition that can make it hard for vision-based gait recognition systems to work [15,16]. (i) First, any variation in walking speed can affect the gait pattern and lead to false identification. (ii) Second, gait recognition can be affected by external factors such as footwear, carrying objects, and, clothing, which can alter the natural gait of an individual. (iii) The presence of occlusion occurs when an object obstructs the view of the walking person, and it can also reduce the accuracy of gait recognition. (iv) Another limitation of gait recognition is its vulnerability to spoofing attacks. Spoofing attacks involve creating artificial gait patterns to mimic the gait of an authorized person and gain unauthorized access. Such attacks can be carried out by using prosthetic limbs, walking aids, or mimicking the walking style of the authorized person. Finally, (v) the major limitation is the effect of changing environmental factors on gait recognition accuracy. Factors such as different lighting conditions, varying camera viewpoints, and different walking surfaces can affect the accuracy of gait recognition. Additionally, the variability of human gait due to factors such as age [17,18,19], health conditions [20], and fatigue [3] can also affect the accuracy of gait recognition.

### 1.1. Gait for Person Recognition

Biometric identification says that gait recognition has a number of similar features that set it apart from other biometric modalities. For instance, gait recognition has important advantages over other biometric systems such as face [2], fingerprint [3], and iris recognition [4]. For example, other biometric systems need access to devices that can take pictures; however, gait information can be collected without the subject’s help because it is not invasive. Video sequences of gait information can be taken from a distance with low spatial resolution. So, gait recognition can identify individuals from a distance based on their walking style, which makes it ideal for applications where it is not possible to be close to the individual being identified [8].

Since the gait recognition system does not require closed subject interaction with the image sensing device, this is highly expected to apply in security and surveillance applications. As walking is one of the main processes for mobility, it is hard for the criminal to disguise the process during walking. In this regard, where other biometric systems are used to identify the suspect in those situations, gait can work [7]. Gait analysis is also used for health monitoring and rehabilitation purposes. For example, gait analysis can be used to detect abnormalities in a person’s walking pattern that may indicate an underlying medical condition. It can also be used to track the progress of rehabilitation after an injury or surgery [21].

In psycho-physiological studies [7,22], it was found that a person’s sex can be guessed with 80% accuracy based on how they walk. It is also revealed that any person’s emotion [23,24], feelings, and body weight [12] can be identified using the gait feature [21]. Most of the time, the approaches used in gait recognition are an end-to-end model, which exclude the preprocessing steps. That is because most of the approaches used for gait recognition learn the human body structure from the analysis of the silhouette or skeleton. Other visual classification issues in computer vision, however, frequently depend on texture-derived features in addition to shape and structure data [25].

Human activity recognition and person re-identification techniques are used to learn representations that capture individual appearance traits that are shared across multiple cameras, such as clothing and skin tone [26]. Instead, gait recognition techniques work to find the right ways to represent walking patterns so that they can be separated from a subject’s appearance and then used to classify them. When gait recognition is compared to human activity recognition techniques [27], the goal of the latter is to find a subject’s specific movements or actions from video sequences, which are called “macro” motion patterns. Gait characteristics, on the other hand, can be thought of as subtle “micro” patterns that rest on top of a particular activity class, namely walking. Therefore, it is frequently more difficult to identify such subtle discriminative information than it is to recognize activities. Additionally, due to the subtlety of gait patterns, which make them distinctive to various subjects, they are frequently influenced by the subject’s current mental state, such as fatigue [21], excitement and fear [28], and even injuries [21].

### 1.2. Data Extraction

To acquire the published papers appropriately from online sources, we followed a procedure by which we searched the papers that were published from 2015 to December 2022 with the keywords “gait”, “gait biometric”, “gait recognition”, “deep learning”, “deep algorithm”, and “neural architecture”. The papers are basically searched online at different digital libraries and Google Scholar, mainly from IEEE Xplore, CVF Library, ACM Digital Library, ScienceDirect, MDPI, arXiv, and SpringerLink.

**Figure 1:**
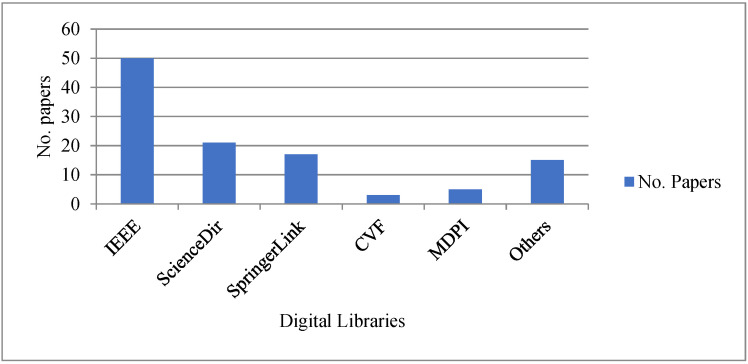
Papers collected for review from different sources. Here, the others mention the papers collected from different journals and conferences, especially from IET, Inderscience, Wiley, and Tailor.

After obtaining the papers through the process of forward and backward searching, we collect the ones that satisfy the aforementioned criteria. Those papers are excluded that are not vision-based, do not mention the new results, and do not use standard as well as private datasets to test and compare with the other methods. The same papers in different libraries are also excluded. The number of papers collected from different sources is presented in Figure 1.

According to Figure 1, the papers collected from IEEE are 45%, where journal papers are 46%, including IEEE-T-MM, IEEE-T-PAMI, IEEE-T-CSVT, IEEE-Access, IEEE-T-IFS, IEEE-T-IP, IEEE-T-CSVT, IEEE-T-Biom, and IEEE-T-NNLS journals. The rest of the IEEE papers are collected from different computer vision conferences, such as IEEE-CVPR, IEEE-ICPR, IEEE-PRCV, IEEE-ICCV, and IEEE-ICPC. Papers collected from science directories and SpringerLink are 18% and 15%, respectively. From MDPI, 4% of papers are collected, especially from electronics, sensors, and Applied Sci.

### 1.3. Background and Motivation

In the COVID-19 pandemic situation, the governments of the globe were taking action to find the virus and stop the outbreak [29]. It required people to wear masks in order to stop the spread, which made it challenging to recognize individuals using the existing, pervasive networks of CCTV cameras. In such a situation, gait analysis can be considered an effective method to identify individuals in a non-intrusive and covert fashion, utilizing the already installed CCTV camera network.

**Figure 2:**
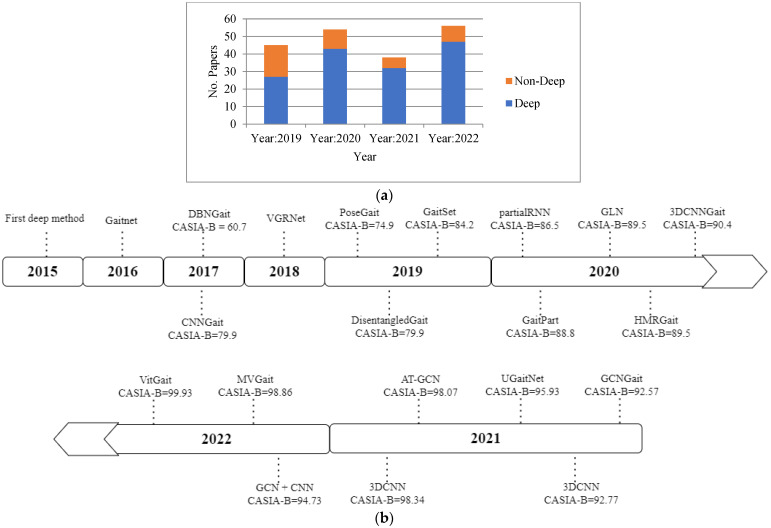
Number of papers published and evaluation process: (**a**) the number of publications published from 2019 to 2022 based on deep and non-deep methods, and (**b**) the evaluation of gait recognition processes based on the CASIA-B dataset from 2015 to 2022, where, first deep method [31] proposed at 2015. At 2016 proposed Gaitnet [32]. BDNGait [33], CNNGait [34] and VGRNet [35] proposed at 2017 and 2018 respectively. At 2019 proposed DisentangledGait [36], GaitSet [37] and PoseGait [38]. At 2020 proposed partialRNN [39], GaitPart [40], 3DCNNGait [41], HMRGait [42], and GLN [43]. GCNGait [44], AT-GCN [45], 3DCNN [29,46] and UGaitNet [47] proposed at 2021. At 2022 proposed GCN + CNN [48], MVGait [49] and ViTGait [50].

**Figure 3:**
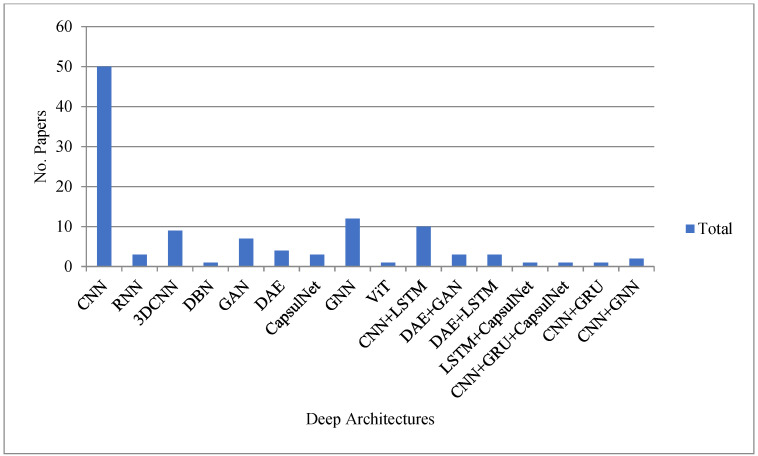
Deep neural architecture methods were used for gait recognition from 2015 to 2022.

In the last decades, the majority of studies in the literature have been published based on vision-based gait recognition that is camera based as opposed to sensor-based or pose-based gait recognition [30]. The previous studies mainly focused on traditional machine learning approaches [8]. However, at the current time, the published works clearly focus on deep learning approaches. The main region behind this is the automatic feature extraction process from the human body representations, i.e., the silhouette or skeleton. The process is also effective because deep learning-based gait recognition is an end-to-end learning process that does not need feature engineering. The number of publications published from 2019 to 2022 based on deep and non-deep gait recognition methods is presented in Figure 2a. From the figure, it is revealed that the research is highly focused on the deep learning-based gait recognition method. The evaluation of the gait recognition process from 2015 to 2022 using the deep method based on the CASIA-B dataset is presented in Figure 2b, which is the most usable dataset for validating the proposed models. From the figure, it is revealed that deep learning-based methods improve person recognition accuracy with time. For instance, the best accuracy of deep methods in 2019 was 84.2%, whereas the best accuracy shown in 2020, 2021, and 2022 was 90.4%, 98.34%, and 99.93%, respectively. The deep learning methods used for gait recognition from 2015 to 2022 are shown in Figure 3.

According to Figure 3, the CNN architecture used the top 45%, while 3DCNN, GAN, LSTM, and GNN used 8%, 6%, 3% and 11%, respectively. For the hybrid structure, CNN + LSTM used 9%. Moreover, DAE + GAN, DAE + LSTM, and CNN + GCN applied 3%, 3% and 2%, respectively. From Figure 3, it is also observed that researchers focused on the different deep learning methods such as CNN, LSTM, 3DCNN, DBN, GAN, DAE, and CapsulNet, and hybrid methods such as CNN and LSTM, DAE and GAN, DAE and LSTM, LSTM and CapsulNet, CNN and GRU, and CapsulNet, highly in the years 2019 and onwards. It is also observed that after 2020, the researchers were focused on the new deep learning methods based on the graph, i.e., the GNN for gait recognition. The fact of this research migration is the updating of human body representation from silhouette to skeleton. After the robust development of human pose algorithms such as OpenPose [51] and AlphaPose [52], the skeleton body representation is robustly improved and overcomes the problems of silhouette-based body representation in gait recognition. The skeleton-based body representation also improved the gait recognition process.

**Figure 4:**
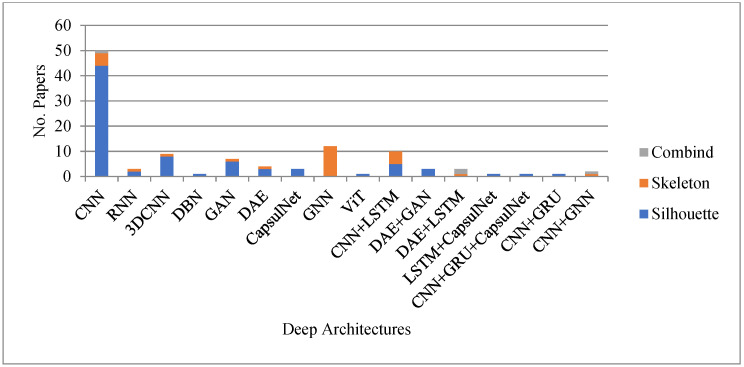
Deep architecture methods used in the different publications according to the body shape representation.

The number of research studies focused on the silhouette and skeleton from 2015 to 2022 is presented in Figure 4. Figure 4 also focuses on the robust gait recognition methods from 2015 to December 2022.

Review papers [16,53,54,55,56,57,58] and [59] have been published about both vision-based and non-vision-based ways to recognize gait. The review papers that have been written about gait recognition using non-vision-based methods focus on the literature up until 2018. Some of the vision-based review papers [7,8,9] look at the literature that has been published up until 2020. Regardless of this, deep learning has recently made a number of significant advancements in the field of gait recognition. To our knowledge, no surveys have concentrated exclusively on the deep learning approach for gait recognition since 2021.

### 1.4. Contributions

The paper aims to provide a comprehensive overview of the advancements and challenges in gait recognition using deep learning methods until December 2022. The paper highlights the potential of gait recognition for identifying individuals based on vision-based approaches. It acknowledges the challenges associated with recognizing gait accurately due to the complexity and variability of environments and human body representations. The paper presents a detailed analysis of various datasets used in the literature review and examines the performance of state-of-the-art techniques. It provides a taxonomy of deep learning methods to organize the research landscape and identifies the limitations of these methods in gait recognition. Finally, the paper suggests research directions to overcome the challenges and improve the performance of gait recognition in the future. Overall, the paper contributes to the field of gait recognition by providing a comprehensive overview of deep learning methods and highlighting the challenges and opportunities associated with gait recognition.

The main contributions of this review paper are as follows:

i. The paper presents a taxonomy of deep learning methods to describe and organize the research landscape in this field. This taxonomy can help researchers and practitioners understand the various approaches and their limitations.
ii. The paper provides a comprehensive overview of the advancements made in the field of gait recognition using deep learning methods.
iii. The paper acknowledges the challenges associated with recognizing gait accurately due to the complexity and variability of environments and human body representations. It also identifies the limitations of deep learning methods in the context of gait recognition.
iv. The paper concludes by focusing on the present challenges and suggesting a number of research directions to improve the performance of gait recognition in the future.

### 1.5. Organization

The organization of this paper has been structured to provide a comprehensive review of gait recognition research. In Section 2, the dataset used in the present state-of-the-art literature is described, which includes information on data collection and the properties of the dataset. Next, a taxonomy of gait recognition methods is presented, which includes an overview of different approaches and techniques used in gait recognition. Trends in gait recognition research are analyzed, and the performance of different methods is evaluated and compared in Section 4. Additionally, the limitations and challenges of gait recognition are discussed in Section 5. After that, research problems and challenges in gait recognition are identified. Finally, the main findings of the paper are summarized, and a list of potential future research directions is presented.

## 2. Datasets

Gait recognition has become a popular way to identify people because it is non-invasive and can work from a long distance. In the past, different datasets have been used to test how well gait recognition algorithms work. These datasets are limited in different ways, such as by the way people look, how they are seen from different angles, and how the environment is. For training, deep structured methods need large datasets with samples, numbers, and environmental conditions that are spread out. In the data preparation process, we have to face two basic problems. One of them is, we have to capture the video or image sequences of an individual during a number of movements within the gait cycle. Another problem is the ethical and privacy issues in public or private spaces for each individual. In this section, we provide a detailed description of the datasets used in gait recognition research. The summary of the datasets that are rapidly used in the different published papers described in this section is presented in Table 1. From Table 1, it is observed that the sequences and view angles of the entire dataset are not the same. The highest view angle datasets are CASIA-B [33], OU-MVLP [60], CASIA-E [61], and OU-ISIR MV [62], and the highest sequence datasets are OU-MVLP [60], OU-ISIR [63,64], and OU-ISIR LP Bag [65].

### 2.1. CASIA-A

CASIA-A [66] is a well-known gait recognition dataset made up of data from 20 people walking in a straight line outside. The dataset was recorded with three cameras placed at angles of 0°, 45°, and 90°. On average, each sequence has 90 frames. For each subject, there are sequences from all three cameras on the training set. The testing set, on the other hand, only has one sequence for each subject that one of the cameras captured. The dataset includes videos captured from different viewpoints, including frontal (0°), lateral (45°), and side (90°), resulting in various poses and walking styles for each viewpoint. Each sequence captures the gait of a single individual, and the sequences have varying lengths, ranging from 4 to 12 s. To evaluate the models’ cross-view recognition performance, the dataset uses a cross-view test protocol, where one camera’s sequence is used for testing and the other two cameras’ sequences are used for training. This protocol ensures that the trained model can recognize individuals from different viewpoints, making it relevant to real-world applications.

### 2.2. CASIA-B

The dataset known as CASIA-B [33] is extensively utilized for gait recognition and features multi-view gait data for 124 individuals in both silhouette and RGB forms. The data were collected from 11 different viewing angles, with 18° increments, covering a range of 0° to 180°. It includes three distinct walking conditions—normal walking (NM), walking with a coat (CL), and walking with a bag (BG)—with six, two, and two gait sequences per individual per view, respectively. For the CASIA-B dataset, in the training phase, we utilize the 74 individuals, and the rest of the samples are used during the testing phase.

### 2.3. CASIA-C

Infrared and silhouette data from 153 different subjects, taken under varying night-time lighting circumstances, are included in the CASIA-C dataset [67]. The dataset contains sequences where the subject is carrying a bag (BW) as well as three different walking speeds: slow (SW), normal (NW), and fast (FW) walking. Per individual, there are 2 FW, 2 SW, 4 NW, and 2 BW sequences. The evaluation process includes exams to identify cross-speed walkers.

### 2.4. CASIA-E

The CASIA-E [61,68] dataset was published in 2020 and used in the published paper last year. It consists of the silhouettes of 1014 individuals with three different scenarios: simple, complex, and complex dynamic backgrounds. Here, for each individual, we provide 100 sequences and three walking variations. The walking variations are normal (NM), wearing coat (CL), and carrying a bag (BG). This dataset was prepared based on the fifteen view angles, including thirteen horizontal views focusing from 0 to 180 degrees with 15-degree intervals. The dataset also includes the two vertical views that were captured at 1.2 and 3.5 m, respectively.

### 2.5. OU-ISIR

The OU-ISIR dataset [63,64] includes images of 4007 subjects’ gaits taken by two cameras at angles of 55°, 65°, 75°, and 85°. The subjects’ ages range from 1 to 94. In the world coordinate system, each angle corresponds to the *y*-axis of the camera’s line of sight (parallel to the walking direction). Each camera angle has a designated bin, and each subject is put in the bin of the camera that caught them. Each subject’s silhouette or GEI features are size-normalized in the collection.

### 2.6. OU-ISIR LP Bag

The OU-ISIR Bag [65] comes from videos of 62,528 people who were inside and carrying things when they were caught on camera. Each individual has three sequences—two with and one without a carried item. For training, the dataset contains 29,097 individuals for both sequences with and without carrying objects. The remaining 29,102 disconnected subjects are part of the test group. For splitting the test data into the probe and gallery, two methods are used: one for cooperative situations and the other for uncooperative ones. The probe set considers seven different carrying objects, whereas the gallery set considers no carrying objects. Both sets are created randomly in an uncooperative way.

### 2.7. OU-ISIR MV

The OU-ISIR MV dataset [62] is a gait dataset with silhouettes of gaits from 168 individuals. Individuals’ ages ranged from 4 to 75, and there were almost equally many male and female subjects. The gait data collection contains measurements made from a number of angles, including 24 azimuth views and 1 top view. Cross-view testing methods have made extensive use of the dataset.

### 2.8. OU-ISIR Speed

The OU-ISIR Speed dataset [69] provides a special collection of gait silhouettes from 34 individuals that are perfect for testing how well gait identification algorithms stand up to various walking speeds. The dataset contains nine different speeds, with an interval of 1 km/h and a range of 2 to 11 km/h. The dataset is a crucial resource for creating and testing new gait recognition algorithms because it uses cross-speed tests to assess how well recognition techniques perform at various speeds.

### 2.9. OU-ISIR Clothing

A special gait dataset, the OU-ISIR Clothing dataset [70], records the gait sequences of 68 subjects wearing up to 32 different kinds of clothing. The dataset was collected inside at two different times on the same day, so the background and lighting were different each time. The dataset has a subject-independent test procedure that separates the data into training, testing, and probe sets. This makes it easier to test how well gait recognition methods work with different types of clothing. In order for the gait recognition techniques to be tested well in hard situations, the testing and probe sets are made to cover every possible combination of clothing and environment. In conclusion, the OU-ISIR Clothing dataset is a very useful tool for researchers who want to figure out how people walk in different clothes. 

### 2.10. OU-MVLP

A large sample size dataset [60], i.e., 259,013 was used for the gait recognition, effectively reducing the overfitting problem that occurs for small samples. This dataset consists of 10,307 individuals with ages ranging from 2 to 87, who are captured from 14 angles. In this dataset, seven cameras are used with an interval of fifteen degrees ranging from 0 to 90 and 180 to 270 degrees. In these intervals, 28 images are captured for each individual. For training and testing purposes, 5153 and 5154 individuals are provided, respectively. Recent published papers consider only four view angles: 0, 30, 60, and 90 degrees, or all view angles.

### 2.11. OUMVLP-Pose

The skeleton-based dataset is created from the OU-MVLP using the two pre-trained human pose estimator algorithms, OpenPose [51] and AlphaPose [52]. The dataset contains information about 10,307 individuals captured from 7 cameras with 14 view angles at an interval of 15 degrees. Each individual gait sequence contains an average of 25 frames. For training and testing purposes, 5153 and 5154 individuals are provided, respectively.

### 2.12. TUM GAID

The TUM GAID dataset [72] is a comprehensive gait dataset made up of RGB, depth, and audio data that were recorded from 305 individuals. The dataset was collected from a subset of 32 people at two separate times in the winter and summer when they were outside. The dataset contains ten sequences for each subject and involves walking normally (N), carrying a backpack (B), and wearing temporary shoe covers (S). The original authors divided the data into training, validation, and test sets and gave a test protocol for the dataset. This dataset is frequently used by researchers to conduct recognition experiments that concentrate on the N, B, and S gait variations.

## 3. Taxonomy

In this section, a taxonomy is used to show a review structure based on deep learning methods. The taxonomy gives an overview of how deep learning is used in different publications and for different lengths of time. Many taxonomies have been proposed in the previous review papers; however, different published papers present different perspectives, such as in [73], where authors explain the taxonomy based on the categories of sensor, covariate factor, and classifier. A feature-based taxonomy is presented in [74]. Another taxonomy based on environmental issues, environmental lighting sources, imaging cameras, and individual appearance is presented in [75]. In [8], the authors proposed a taxonomy that highlights the different classifiers, such as deep learning-based and traditional-based. Finally, in [9], there is a proposed taxonomy that is separated into four parts. The parts are body representation, temporal representation, feature representation, and neural structure. This paper draws inspiration from [9] and proposes a taxonomy of deep learning techniques to describe and arrange the research landscape in this area. This paper also identifies the limitations of these methods in gait recognition and provides research directions to overcome the challenges and improve the performance of gait recognition in the future.

In the process of gait recognition, different deep learning methods use different deep architectures, such as convolutional neural networks (CNNs) [31], long short-term memory (LSTM) [22], 3DCNN [76], Deep Belief Network (DBN) [77], Generative Adversarial Network (GAN) [78,79], Deep Auto Encoder (DAE) [80], capsule networks (CapsNets) [81], graph neural network (GNN) [82], and different hybrid methods, to automatically extract features from the shapes of the human body. Some of the literature uses different deep architectures together to extract efficient features, including CNN + RNN [83], DAE + GAN [74,84], DAE + RNN [36,85], RNN + CapsNet [81], and CNN + GNN [48,86]. The published papers basically present the body shape in two different ways: one is appearance-based, which is the silhouette, and another is pose-based, which is the skeleton: the 2D or 3D body joint representation [87].

**Figure 5:**
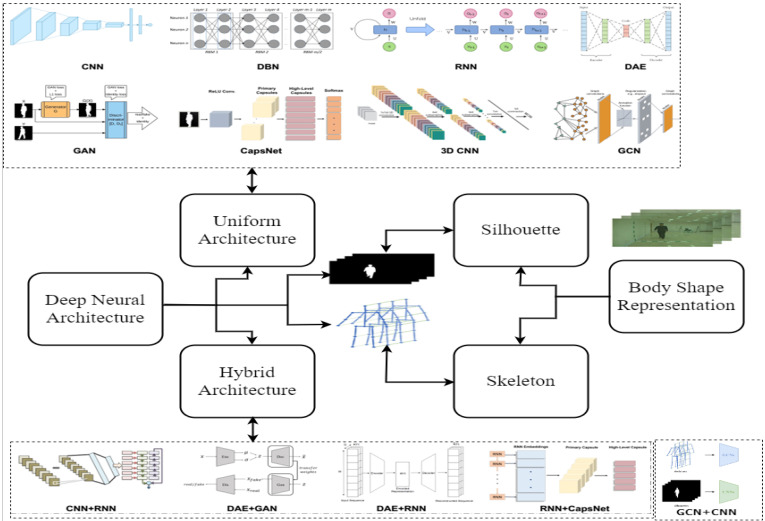
The proposed taxonomy: applying the deep learning architectures on the different body shape representation.

Based on the aforementioned concept, the proposed taxonomy is split into two main groups: uniform deep architecture and hybrid deep architecture. Each category is further divided into two parts that are the two ways the human body shape is represented, i.e., silhouette and skeleton. The deep learning architecture’s performance and limitations are focused on the human body’s shape. For example, the appearance-based representations have state-of-the-art limitations such as silhouette images that create some disparity problems for a person’s covariate factors and viewpoint changes that degrade the performance of the gait recognition. However, skeleton-based human body shape representation recovers these issues and improves the performance of the gait recognition process with deep architectures. Furthermore, much of the deep learning-based architecture has limitations for skeleton-based body representation. All of these will be mentioned and explained in the following sections. The representation of the proposed taxonomy is shown in Figure 5.

### 3.1. Uniform Deep Architecture

Uniform deep architectures are the single deep architectures used uniformly to extract the abstract features from the gait-based body representations, such as a silhouette or skeleton, to identify the gait steps in the gait cycle. Since 2015, different deep neural architectures have been utilized in the field of gait recognition and have achieved significant improvements in this field. The deep architectures utilized in the different publications that contribute to improving the performance of gait recognition based on camera vision are explained here.

#### 3.1.1. Convolutional Neural Network (CNN)

Deep learning algorithms known as convolutional neural networks (CNNs) [82] are frequently used for feature extraction in computer vision tasks, including gait recognition [88,89,90,91,92]. CNN performs the convolved operation on images to extract the abstract features from the spatial dimension in a hierarchical manner [93]. In gait recognition, CNN-type models are utilized to embed the silhouette or skeleton body shape or structure in the spatial feature space.

CNN works best when it is set up in the best way, which is a mix of convolutional, pooling, and fully connected layers. CNNs work by applying a set of learnable filters, also called kernels or weights, to the input data in a sliding window fashion. These filters are made to find certain patterns or parts in any structure, such as the shape of a body. They also include activation functions such as ReLU [94] or Tanh [95] to increase non-linearity. Pooling layers use non-linear down-sampling strategies, such as average or maximum pooling, to reduce the spatial size of the feature maps and decrease network complexity. Finally, fully connected layers transform the resulting two-dimensional feature maps into one-dimensional vectors for further processing.

By analyzing the present CNN methods published in the different publications, it is observed that researchers utilized the shallow neural network, whereas different applications utilized the deep neural network for improving performance. For a better understanding of the scenarios and why researchers utilized the shallow neural network, here, we summarize some of the CNN architectures published in different publications based on the convolutional layer, pooling layer, and fully connected layers. The input dimension is also considered in the summarization. In this summarization, we only consider the CNN structure, ignoring the other embedded architectures such as the method with CNN and LSTM. The summary is presented in Table 2 [39].

From Table 2, it is revealed that the highest range of layers is from six to sixteen. However, if we carefully look at Table 2, it is observed that the significant CNN models have six to ten layers in combination. The input dimensions of the CNN models are 64 × 64, 88 × 128, 120 × 120, and 128 × 128. In [97], the Gait-Part model shows significant improvement for the CASIA-B dataset with an accuracy of 96.70%. This model just uses nine layers, and the input dimension is 64 × 64. In [34], Ensem-CNNs justified the performance of the CNN models for the different input dimensions and with the same layers. From the literature, it is observed that the 64 × 64 input dimension shows improved results. The reason behind that is that the higher-dimension input models need more layers for extracting significant features. As a result, the 64 × 64 input dimensions are widely used to reduce computational complexity.

The main reason to use the lower layers in the CNN model is the end-to-end model. In the gait recognition process, we fit the silhouette or skeleton image into the CNN model. From there, the CNN models can extract the features of gait, body shape, and steps features effectively. As the absolute human body shape is used in the CNN model, no preprocessing is required here. As a result, the model is going to be simplified and can represent the significant features of the human body shape, such as a silhouette or skeleton.

#### 3.1.2. Generative Adversarial Networks (GAN)

Generative adversarial networks (GANs) are a type of deep learning algorithm made up of two neural networks: a generator and a discriminator [79]. The generator makes fake samples that look like the real data, and the discriminator learns to tell the difference between the real and fake samples. Through this adversarial process, both networks learn and improve their abilities to generate and distinguish between real and fake samples [78,105].

GANs have been recently applied to gait recognition [40,74,102,106,107,108,109], where they are used to generate synthetic gait data to augment the training dataset. This is particularly useful when the available dataset is small or imbalanced, as GANs can generate diverse and realistic synthetic data to balance the dataset. In addition, GANs can also be used to generate data from different viewpoints or under different conditions, allowing for better generalization of the model. In this regard, GAN is applied in the gait recognition process, where body representation is a silhouette. As GAN has the ability to handle viewpoint changes and manage the disparity between the different human appearance representations, it would be a suitable choice for gait recognition.

However, there are also limitations to using GANs for gait recognition. One of the main challenges is the quality of the generated data, which may not always be realistic or diverse enough to improve the model’s performance. In addition, GANs require a large amount of computational resources and may be difficult to train and fine-tune for optimal performance. Nonetheless, GANs have shown promise in improving the performance of gait recognition models and are an area of active research.

In the recently published papers, different GAN architectures are utilized for gait recognition [40,77,79,102,104,105,106,107,108,109]. One of them is MGGAN [102], which is the multi-task GAN focus for overcoming the limitation of cross-view gait recognition in different environmental conditions. Here, CNN architecture is applied to extract the human view of specific body representation features in the spatial space; after that, one view to another is transformed using a transform layer, and the process learns the temporal information of gait steps. The network is learned by pixel-wise loss and multi-task adversarial techniques. Another GAN-based method, namely DIGGAN [74], is used for gait recognition. Here, the GEI is transferred to a different perspective to identify the gait information. For that, two discriminators are utilized. TSGAN [106] is proposed for gait recognition with cross-view angles. The TSGAN is used here to change the perspective of the GEI’s temporal viewpoints. The two streams of GAN learn the temporal and spatial features from the GEI automatically.

#### 3.1.3. Deep Belief Networks (DBN)

Deep Belief Networks (DBNs) have also been used for gait recognition. In a study [77], a DBN was trained to learn a hierarchical representation of gait features, which was subsequently used to identify individuals from gait sequences. The DBN was composed of a stack of Restricted Boltzmann Machines (RBMs) [81], which were trained in a layer-wise manner to learn increasingly complex representations of the gait data. The resulting deep features were then fed into a classifier for person recognition.

DBNs are better than traditional shallow networks because they can learn more abstract and complex representations of data [110]. This may be helpful for gait recognition, where it can be hard to pick up on small differences between people’s steps. However, DBNs require more data and computational resources for training than shallow networks, and they may also suffer from issues such as vanishing gradients during training. Many DBNs are utilized for person identification using gait [110,111]. The research presented in [110] focused on extracting fitting body parameters and shape features from the silhouette. These features were then learned by Deep Belief Networks (DBNs) to extract more discriminative gait features. Similarly, in [111], gait was represented as motion and spatial components, which were then used to train two separate DBNs. Finally, the extracted features from each DBN were concatenated to form the final feature representation for gait recognition.

#### 3.1.4. Capsule Networks (CapsNets)

Capsule networks (CapsNets) are a relatively new type of neural network that has shown promise for many computer vision tasks, including gait recognition [112,113,114]. In a paper [81], Hinton et al. introduced CapsNets. In CapsNets, the basic processing unit is called a capsule, which can be thought of as a group of neurons that represent a specific instantiation parameter, such as pose or deformation. Capsules are organized in layers, and each layer can be thought of as a set of capsules that vote to determine the properties of higher-level capsules in the next layer. In gait recognition, CapsNets have been explored as an alternative to CNN-based approaches. One advantage of CapsNets is that they can capture the spatial relationships between different parts of a silhouette or skeleton image, which can be useful for recognizing complex patterns such as gait [81,112].

CapsNets have been applied to gait recognition in various ways. For example, CapsNets were used to learn the spatial relationships between body parts in gait videos. Moreover, a CapsNet was trained to learn the 3D structure of the human body from RGB-D data and use this information for gait recognition. Compared to traditional CNNs, CapsNets have shown advantages in dealing with viewpoint changes and data variability, and they have the potential to capture richer spatial relationships between body parts. However, the high computational cost of CapsNets remains a limitation for real-time applications [115]. However, one limitation of CapsNets is that they can be computationally expensive and may require more training data compared to CNNs. Additionally, the interpretability of CapsNets can be challenging, as the outputs are represented as vectors of probabilities rather than feature maps. The benefits of CapsNets are adopted for recognizing individuals based on the gait analysis [112,113,114]. In [112], we proposed a method to recognize a person based on gait. For that, initially, we apply the CNN to the GEI to extract the properties of templates. After that, the dynamic routing of the CapsNet is applied to retain the temporal information between the templates and extract robust spatial–temporal features. In [113], we proposed a method to focus on extracting discriminative features from the GEI image with different covariate factors. Here, two capsule networks are utilized. The first one is used for extracting the bottom layer’s features by matching with another capsule network. A second capsule network is used to extract the features from the middle layers. This method shows effectiveness for cross-view angles, cross-walks, and clothing. In [114], the researchers present another capsule network for gait recognition where a pair of GEIs is used. Here, the gait features are extracted using the CNN and provide an effective output feature by using the similarity of the image pair through the capsule network. 

#### 3.1.5. Recurrent Neural Networks (RNNs)

Recurrent Neural Networks (RNNs) [116] are a type of neural network architecture that can process sequential data by retaining information in its hidden state. RNNs have been used for gait recognition as well, where the sequence of gait data is fed into the network and the hidden state of the network is updated at each time step based on the current input and the previous hidden state. The hidden state thus retains information about the previous inputs in the sequence and allows the network to learn temporal dependencies between different frames.

In gait recognition, RNNs have been used to process different types of gait data, such as silhouette, joint angles, and acceleration signals [15,60,78,102,111,117,118]. For example, an RNN can be used to process the silhouettes of different gait cycles, and the learned features are then used for classification. Moreover, an RNN can also be used to process joint angles of different gait cycles, and the learned features were used for gender classification [87,119]. It can be used to process acceleration signals from wearable sensors, and the learned features are used for activity recognition. RNNs have the advantage of being able to capture long-term temporal dependencies in the gait data, making them suitable for tasks such as activity recognition or gait analysis over a longer time span. One limitation of using RNNs for gait recognition is that they can suffer from the vanishing gradient problem, which can make it difficult to learn long-term dependencies [15,120]. Additionally, RNNs can be computationally expensive, making them less suitable for real-time applications.

For overcoming these problems, different structural LSTM [111] and GRU [15] are used. These are the ways the RNN process can maintain the relationship among the gait sequences with memory and learnable function. The LSTM network [22] uses cells that have a shared cell state to hold long-term dependencies using input and forget gates all the way down the chain of LSTM cells. These gates give the network the ability to determine when to discard the previous state or add new data to the current state. An output gate controls each cell’s secret state, or output, which is calculated based on the most recent cell state. On the other hand, unlike the LSTM, the GRU [15,121] is a kind of RNN that does not employ the output activation functions. It has an update gate that allows the network to modify its present state in response to fresh data. The output of the gate, also referred to as the reset gate, only keeps links with the cell input.

**Figure 6:**
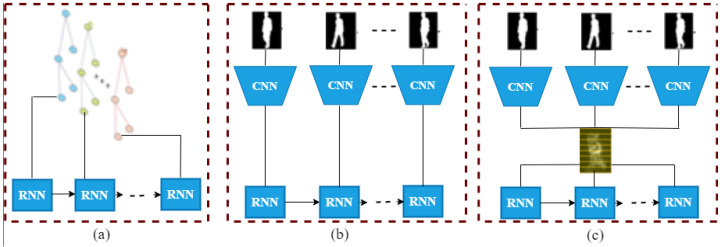
Different RNN approach: (**a**) RNNs recurrently learn the relationships between partial representations in gait templates, (**b**) CNNs and RNNs are merged, and (**c**) RNNs directly learn from the movement of joint positions [9].

RNNs can be used in one of three ways to recognize gaits. The first way, which is typical for skeleton representations and is shown in Figure 6a, is to use RNNs to learn from how the locations of joints change over time. RNNs are combined with other neural architectures, especially CNNs, in the second method (illustrated in Figure 6b) [15,118], which will be covered in more detail in the hybrid section, to learn both spatial and temporal information. The third strategy—adopted lately in studies such as [39] and [115]—involves using RNNs to repeatedly learn the connections between partial representations drawn from a single gait template, such as GCEM [39].

#### 3.1.6. Three-Dimensional Convolutional Neural Networks (3DCNN)

Three-dimensional convolutional neural networks (3DCNNs) have been recently applied to gait recognition due to their ability to capture both spatial and temporal feature information over the full gait cycle [41,76,117,122,123,124]. In 3DCNNs, the convolutions are performed along the spatial as well as the temporal dimensions, which enable them to learn spatiotemporal patterns directly from video sequences [29]. The process is more robust for vision-based viewpoint changes and disparity issues in the subject’s appearances. One of the challenges of using 3DCNNs for gait recognition is the high dimensionality of the input data, which requires significant computational resources. However, this issue can be addressed by using techniques such as early fusion, in which spatial and temporal information are combined before feeding the data into the network. Another limitation of 3DCNN is its inflexibility in processing varying-length sequences for gait detection. These limitations are addressed in [41]. The problem is overcome by introducing the hybrid 3DCNN for integrating temporal discriminative features on different scales. In [76], a research study suggested 3DCNN architecture for recognizing gaits. The network had two completely connected layers, 13 3D convolution filters, and pooling layers. The standard 3D pooling layer was changed in another approach described in [124]. The process is accomplished by combining global and partial 3D convolutional layers with local clips to aggregate temporal information.

#### 3.1.7. Deep Auto Encoders

Deep Auto Encoders are a type of neural network architecture that has been used for gait recognition [80,125,126]. The process involves training the network to encode input gait data, such as images or motion sequences, into a lower-dimensional representation or code. This code can then be used as a feature for classification or clustering tasks. It is basically an encoder–decoder process, where the encoder represents the bottleneck feature in latent space and the decoder represents the original input data through the opposite operation, which is the stack of convolutional layers.

One of the advantages of using Deep Auto Encoders for gait recognition is that they can learn useful and discriminative features without requiring labeled data. This can be particularly useful in scenarios where obtaining labeled data is challenging or expensive. Additionally, the lower-dimensional representation learned by the network can often be more robust to variations in the input data, such as changes in clothing or lighting [126]. However, Deep Auto Encoders also have some limitations when used for gait recognition. For example, the quality of the learned features can be highly dependent on the architecture and hyper-parameters of the network as well as the quality and quantity of the training data. Additionally, they may not be as effective at capturing the temporal dynamics of gait as other types of neural networks, such as RNNs or 3DCNNs. Some DAE methods are used for gait recognition [60,80,125,126]. According to the process presented in [125], latent features are estimated using the DAE architecture through the four consecutive convolution layers. To reverse the convolutional input, four de-convolutional layers are utilized. To extract the robust gait feature from DAE, seven linked convolutional layers are used in [80]. Another method used by Google LeNet is the inception module [60]. Here, in the decoder, the multi-scale discriminative and covariate features are estimated. These features then fit into the decoder with de-convolutional layers to recreate the temporal template.

#### 3.1.8. Graph Convolutional Networks

Graph Convolutional Networks (GCNs) [82] are a type of neural networks that are designed to work with graph-structured data. In the context of gait recognition, the human skeleton can be represented as a graph, where joints correspond to nodes and the connections between them correspond to edges. One of the advantages of using GCNs for gait recognition is their ability to model the spatial relationships between the joints in the skeleton. They can also take temporal information into account by processing sequences of graphs. This makes GCNs a suitable choice for recognizing gaits with varying speeds and styles [49,127,128].

However, a limitation of GCNs is that they require the graph structure to be known in advance. This can be problematic in scenarios where the data are noisy or incomplete. Another limitation is that GCNs may not perform well when dealing with large graphs, as the computation and memory requirements can become prohibitively high [44,129].

Despite these problems, GCNs have shown promise for recognizing gait, with a number of studies showing state-of-the-art performance on benchmark datasets. The GCN methods are used to overcome the limitations of silhouette-based body representations. Several methods are used starting in 2019, when the human pose estimation process is performed robustly. As such, the literature [104] builds a spatiotemporal graph from the viewable video frames in order to extract gait characteristics. Using a joint relationship learning method, the features are mapped onto a more discriminative subspace with respect to the human body structure and walking behavior.

### 3.2. Hybrid Deep Architecture

Hybrid networks for gait recognition use different kinds of neural networks to do gait recognition tasks better. By taking advantage of the best parts of different neural network architectures, hybrid networks can navigate around some of the problems with single networks and make gait recognition more reliable and accurate.

Combining CNN and RNN architectures is an example of a hybrid network used for gait recognition. CNNs are good at learning spatial features from image data, while RNNs are great at figuring out how events in a sequence depend on each other in time [83]. By combining the two, the hybrid network can learn both spatial and temporal features for gait recognition. Another instance is that by combining a DAE network and CNN architecture, the DAE network extracts bottleneck features from the input gait data, which are then used as input to the CNN. The CNN then learns spatial features from the bottleneck features extracted by the DAE network. Hybrid networks for gait recognition can offer several advantages, such as improved performance, better generalization to different conditions, and more robust feature extraction. However, designing and training hybrid networks can be more complex and time consuming than individual networks, and the resulting network architecture may be more difficult to interpret.

For improving the accuracy and overcoming the limitation of uniform architecture, several hybrid deep architectures are utilized for recognizing the person using gait analysis. The hybrid deep structures are: CNN + RNN (CNN + LSTM; CNN + GRU), DAE + GAN, DAE + RNN (DAE + LSTM), RNN + CapsNet (CNN + GRU + CapsNet; LSTM + CapsNet), and CNN + GNN.

#### 3.2.1. CNN + RNN:CNN + LSTM and CNN + GRU

CNN + RNN hybrid networks have shown promising results in gait recognition tasks by leveraging both spatial and temporal information [130]. The convolutional layers extract spatial features from each frame of the gait sequence, while the recurrent layers process the temporal dependencies between frames. The CNN + LSTM network is proposed for gait recognition, where the CNN layers extract spatial features from each frame of the gait sequence and the LSTM (long short-term memory) layer captures the temporal dependencies between frames [131]. The final output of the network was fed into a fully connected layer for classification. The CNN + GRU network is proposed, where the CNN layers extract spatial features and the GRU layer processes temporal dependencies [39]. The output of the GRU (gated recurrent unit) layer was fed into a fully connected layer for classification. Overall, the combination of CNN and RNN allows for better feature extraction and modeling of temporal dependencies, resulting in improved gait recognition performance.

LSTM and GRU are commonly used instead of traditional RNNs for gait recognition because they can better handle the problem of vanishing gradients that is common in training RNNs [39]. The vanishing gradients problem happens when the gradients used to update the weights in back-propagation become very small. This makes the network learn slowly or not at all. LSTM and GRU networks, which use gates to regulate information flow through the network, provide a solution for this issue. These gates give the network the ability to choose what information to remember or forget over time. This lets them model long-term dependencies in the input sequence. This is especially helpful for gait recognition because it lets the network see the temporal patterns and dependencies in the gait data over time. This makes recognition more accurate.

A deep gait detection system is proposed in [83] that utilizes LSTM and eight distinct CNN architectures to extract spatiotemporal features from image sequences. Another method based on silhouette is proposed in [130], where the silhouette image is divided into four horizontal parts. Then, each part is fed to a separate CNN with ten layers. The output of frame-level attention ratings for each sequence of CNN features was then produced by an attention-based LSTM. The final step was to multiply the CNN features by their respective weights in order to concentrate only on the key frames for gait recognition. In [39], an eight-layer CNN was used to train convolutional maps from gait frames. The GCEM templates were created by combining the convolutional maps and splitting them into horizontal segments. An alert bi-directional GRU learned these incomplete features (horizontal bins) in order to take advantage of the relationships between these embedding components.

#### 3.2.2. DAE + GAN

Deep Auto Encoder (DAE) and Generative Adversarial Networks (GANs) have been used together for gait recognition in some recent works [74,84,109,132]. In this approach, DAE is used to learn compressed representations of gait sequences, and GAN is used to generate new samples based on these compressed representations. For instance, a gait recognition framework based on DAE and GAN is utilized. In this work, the DAE was used to learn a low-dimensional representation of gait sequences, which was then used to train a GAN to generate new samples. The generated samples were used to augment the training data, which improved the performance of the gait recognition system. Overall, the combination of DAE and GAN has shown promising results for gait recognition, especially for data augmentation and cross-view recognition. However, there are still some challenges in training these models, such as finding the right balance between reconstruction error and adversarial loss, and avoiding mode collapse in the GAN training. In [74] and [84], two approaches are presented, namely GaitGAN and GaitGANv2, with the encoder and decoder architectures for discrimination as well as identification of fake and real. That ensured the generated gait images were realistic and contained discriminative information. Another name for this method is alpha-blending GAN, i.e., AbGAN [109]. It creates gait templates using an encoder and decoder network as a generator without original object information. Furthermore, cycle consistent attention-based GAN, i.e., CA-GAN [132], is introduced to synthesize the gait view. Here, the encoder and decoder structures present two branches for exploiting the global and partial discriminative features simultaneously.

#### 3.2.3. DAE + RNN: DAE + LSTM

The combination of Deep Auto Encoders (DAEs) and recurrent neural networks (RNNs) has been explored in gait recognition research [36,85,133]. In this approach, the DAE is used to extract bottleneck features from the gait sequence, which are then fed into an RNN for temporal modeling and classification. The RNNs used are typically long short-term memory (LSTM) or gated recurrent unit (GRU) networks, which can capture long-term temporal dependencies and handle variable-length input sequences.

By using this method, it is clear that the DAE is used to extract features from each gait cycle of a walking sequence, and an LSTM is used to learn the temporal dynamics of the sequence in order to classify it. The results showed that the approach outperformed other state-of-the-art methods on a benchmark gait recognition dataset such as [133] and showed excellent performance on the CASIA-B [33] and OU-MVLP [60] datasets. Another DAE + GRU hybrid network for gait recognition may apply, where the DAE is used to extract bottleneck features, which are then fed into a GRU network for temporal modeling and classification. The GRU network was designed to capture both the short-term and long-term dynamics of the gait sequence. Overall, the DAE + RNN approach has shown promising results in gait recognition and has the potential to capture both spatial and temporal information in a gait sequence for improved recognition accuracy.

The strategy involved separating gait features, such as identity information from appearance, and canonical features that hold irrelevant information for gait recognition using a deep encoder–decoder network and novel loss functions. Once the temporal dynamics had been captured by the resulting gait features, they were fed into a multi-layer LSTM to be aggregated for identification reasons [36,133].

#### 3.2.4. RNN + CapsNet:CNN + GRU + CapsNet and LSTM + CapsNet

Gait recognition using RNN + CapsNet involves using a recurrent neural network (RNN) to capture the temporal dynamics of gait sequences and a Capsule Network (CapsNet) to extract pose and spatial relationship information [81]. In a hybrid RNN-CapsNet network for gait recognition, the output of the RNN is fed into the CapsNet to obtain the final classification result. The CapsNet is used to extract more discriminative features, and its dynamic routing mechanism helped to model the spatial relationships between different body parts during walking [114]. The process achieves promising results on benchmark datasets, demonstrating the effectiveness of using both RNN and CapsNet for gait recognition with different view and appearance changes. Additionally, CapsNet, which can function as an attention module, gives more attention to the important characteristics features.

Combining convolutional neural networks (CNNs) with recurrent neural networks (RNNs) and capsule networks (CapsNets) has shown promise for gait recognition [115]. In the gait recognition process, a CNN is used to extract spatial features from the gait silhouette, which are then fed into a gated recurrent unit (GRU) to capture temporal information. The output from the GRU is then passed through a CapsNet to obtain the final gait recognition results.

In another way, an LSTM is used instead of a GRU to capture the temporal dynamics of gait features. The LSTM’s output is then fed into a CapsNet to obtain the final results for recognition. Overall, these studies suggest that combining CNNs with RNNs and CapsNets can effectively capture both spatial and temporal information in gait sequences, leading to better recognition performance.

In the research described in [115], a CapsNet was used to store the partial representations of a convolution template that were learned over and over again as capsules. This made it possible to learn the coupling of weights between partial features. This method made it easier to generalize to gait views that were not seen during testing. It did this by using relationships between partial features while keeping their positional features. While this was going on, [134] took advantage of the spatial and structural connections between body parts using a capsule network with dynamic routing. Before being put into the capsule network, the recurrent features were taken from a series of gait frames using an LSTM network. 

#### 3.2.5. CNN + GNN

CNN + GNN, also known as the graph convolutional neural network (GCNN), is a deep learning architecture that combines the power of convolutional neural networks (CNNs) and graph neural networks (GNNs) [48,86,91]. This architecture is used for gait recognition and has shown promising results in recent studies. In this context, a CNN is used to extract spatial features from gait images, while a GNN is used to model the spatiotemporal relationships between the extracted features. Specifically, the gait sequence is first represented as a graph, where the nodes represent the extracted spatial features from the gait images and the edges represent the relationships between these features. The GNN is then used to propagate information between the nodes and aggregate the features in a way that captures the spatiotemporal relationships between them. Finally, a fully connected layer is used to classify the gait sequence.

The benefit of using CNN + GNN to recognize a person’s gait is that it can record both the location and timing of the sequence of steps. The CNN is good at pulling out spatial features from gait images, while the GNN can model how the features relate to each other in space and time. However, the hardest part of using CNN + GNN is coming up with a graph structure that can show how the features are related to each other. The hybrid representation of CNN and GNN can reduce the problems with CNN that occur during its uniform use. For instance, CNN treats the skeleton as a grid-shaped structure, whereas the skeleton is a non-Euclidian distance graph-shaped structure.

In [86], two stream-based gait recognition methods are presented, namely graph and image-like representations. The graph-like representation is used in the GCN, and the image-like representation is used in the CNN to extract the features for the event stream. These two streams are called EV-Gait-3D-Graph and EV-Gait-IMG. In another study [91], the authors focused on reducing the problem of the hard sample issue, where the same pedestrian shows a distinct silhouette, and a different silhouette can show the same pedestrian. For that, memory-augmented progressive learning (Gait-MPL) is used to tackle the hard sample issue. Gait-MPL is composed of two processes: dynamic reweighting for progressive learning and a globally structured aligned memory bank. Because the silhouette and skeleton are both effective ways of representing gait appearance, in [48], he proposes a new method where features from both models are extracted by CNN and GCN, respectively. In GCN, a new fully connected GC operator is used. However, the performance of this operator is not satisfactory yet. Later, the STC-ATT module is used for extracting spatial, temporal, and channel-wise information simultaneously.

## 4. Trends and Performance Analysis

This section presents an overview of current gait recognition trends, focusing on the effectiveness of various deep methods and datasets used in various studies in the literature. The analysis is based on publications related to body shape and emphasize recent developments. The description is performed according to our taxonomy and presented in Table 3.

### 4.1. Body Shape

**Figure 7:**
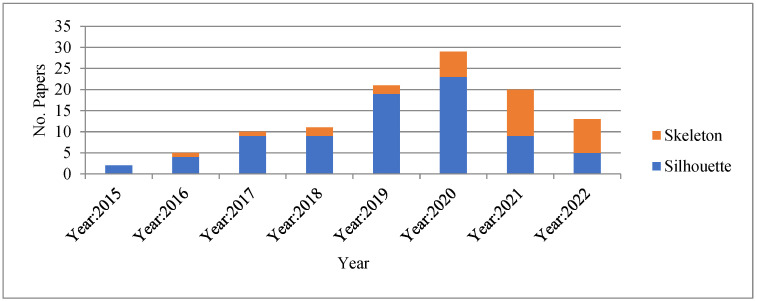
Different body shape used in the different publications according to the publishing year.

The shape of the body is an important part of gait recognition because it can tell a lot about how a person walks. These informational features can change the way a person walks and can also be used to tell one person from another. The present state-of-the-art methods used body shapes in two basic categories: silhouette-based or skeleton-based representations for gait recognition, where the silhouette is the appearance and the skeleton is the model-based representation. From the analysis of Table 3, it is observed that at the early stage of the deep methods, researchers utilized the silhouette-based body shape; however, after 2020, researchers focused on the skeleton-based representation. The percentage of silhouette and skeleton body shapes used in different publications is presented in Figure 7.

From Figure 7, it is observed that until the year 2020, most of the publications chose silhouette-based body representation; after 2020, skeleton-based body representation is used. The reason is the limitation of silhouette-based body shape for gait recognition processes and the advancement of human pose estimation processes such as OpenPose [51] and AlphaPose [52]. As a result, from 2021 on, the majority of model-based gait recognition methods have focused on skeleton based deep neural network architectures.

From Table 3, it is revealed that 70% of publications used silhouette-based body representation and 24% of publications used skeleton based-body representation. Moreover, it is also observed that 7% of publications used both silhouette and skeleton-based body representation. While skeleton-based body shape representation overcomes the limitations of silhouette-based body shape representation and achieves significant improvements in recent years, the skeleton also has limitations, such as occlusion, that are recovered by the silhouette-based representation. For that reason, we anticipate that in the future, models based on the combination of silhouette and skeleton would gain popularity.

#### Datasets

**Figure 8:**
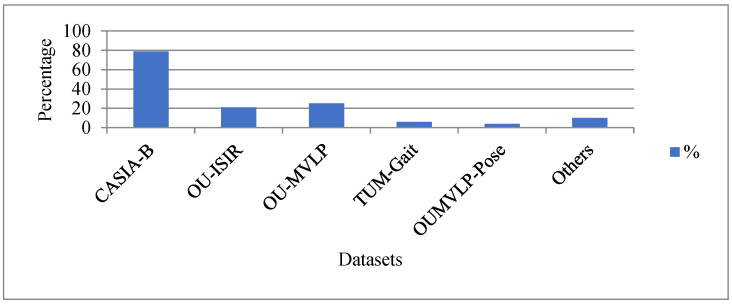
Frequency of dataset used for gait recognition (in %).

According to Table 3, the frequently used datasets for gait recognition are CASIA-B [39], OU-ISIR [63,64], OU-MVLP [60], TUM-Gait [72], and OUMVLP-Pose [71], which are 79%, 21%, 23%, 6%, and 4%, respectively. The other datasets, such as CASIA-A [66], CASIA-C [67], OU-MVLP-Bag [65], and CASIA-E [61,68], are used for 10%. The percentage of the dataset used for gait recognition is presented in Figure 8. From Table 3, it is observed that as of 2021, the significant number of published papers used the CAEA-B, OU-MVLP, and OU-MVLP-Pose datasets to validate their methods, with a percentage of 91%, 38%, and 7%, respectively. Since the year 2020, the CASIA-E dataset has gained popularity due to its diversity. However, the dataset is only used in the literature where body shape representation is in silhouette. We anticipate that this dataset (CASIA-E) will become the standard benchmark dataset for silhouette and OU-MVLP as well as CASIA-B for skeleton-based gait recognition in the future.

### 4.2. Performance of Deep Methods on Datasets

To present the performance of the published deep architectures, we only consider the two datasets CASIA-B [33] and OU-MVLP [60], as these datasets are mostly used from the year 2021. The performance of the literature validated with the CASIA-B dataset is presented in Table 4. On the CASIA-B dataset, paper [90] shows the best recognition rate until the year 2019. At the present state of the art, paper [50] shows the best recognition result. However, the paper does not show the performance for Normal walk; Carrying bag; or Walk with wearing coat individually; it just provides the average recognition accuracy. Based on the overall performance evaluation, the literature [41] shows the best result for the year 2020, which is 90.40%. Some methods [29,45,47,100], and [173] produced outstanding results for gait recognition on CASIA-B in 2021. However, the paper [45] shows superior performance. The recognition accuracy of this paper is 98.07%, which is the best accuracy on the CASIA-B dataset based on the overall performance evaluation. The performance result of the present literature based on the OU-MVLP is presented in Table 5. The best performance of deep methods on the OU-MVLP until 2020 was 89.18%. Some methods [73,88,98] produced outstanding results for the gait recognition rate on OU-MVLP in 2021. Among the methods, ref. [98] shows the best performance on the OU-MVLP dataset, which is 98.00%. Ref. [90] shows a better result in in 2022, which is 96.15%. The method [90], however, does not outperform the method [98] published in 2021.

## 5. Limitations and Challenges

**Figure 9:**
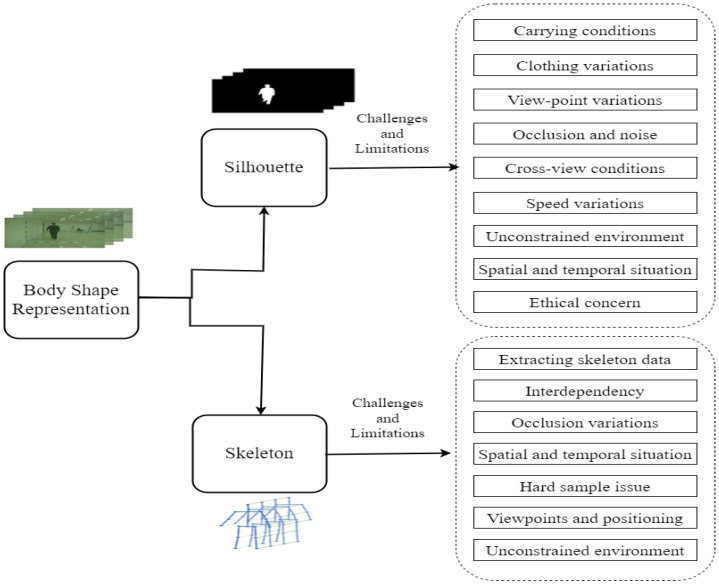
Different covariate issues affect the gait recognition system.

Gait recognition has received significant attention in recent years due to its potential applications in various fields, including surveillance, healthcare, and biometric identification [48,127]. However, despite the growing interest and advancements in gait recognition, there are still several challenges and limitations that need to be addressed. One of the main challenges is the significant variation in gait caused by individual differences, clothing, carrying conditions, and walking speeds [49,174,177]. Additionally, the quality of the input data, such as the resolution, illumination, and occlusion, can significantly affect the performance of gait recognition systems. Furthermore, the ethical and legal considerations related to the use of gait recognition, such as privacy violations and misidentification, should be considered [8]. Therefore, developing robust and accurate gait recognition systems that can overcome these challenges and limitations is crucial for the successful implementation of gait recognition in real-world applications. The different covariate issues that affect gait recognition accuracy are presented in Figure 9.

The gait recognition model basically represents two ways: model-free, which basically focuses on silhouette, and model-based, which focuses on skeleton. The limitations and challenges of gait recognition methods are explained below.

### 5.1. Model-Free-Based Limitations and Challenges

A model-free approach focused on silhouette shape and the dynamic information that is required for gait pattern matching. The silhouette is independent of video quality, which makes the recognition system capable from a distance in a non-invasive and non-intrusive manner. These properties make the system capable of working in the surveillance system. The main limitation of this approach is the covariate facts. In gait recognition, the main challenge is to identify the unknown covariate that has the most impact on the training and testing of a specific person.

The main covariate factors that affect the accuracy of gait recognition are presented here.

#### 5.1.1. Carrying Conditions

Carrying objects during mobility may change the walking pattern as well as the person’s body structure. During mobility, carrying an object may change the walking pattern. So, in those situations, the gait recognition method is not able to provide the accuracy needed.

#### 5.1.2. Clothing Variations

People wear different types of clothing in different environments and seasons. As a result, the body shape with different clothes, such as T-shirt, coat, or shirt, will be different. Moreover, tight and bulky clothing may change the person’s mobility and have an impact on gait recognition. Furthermore, heavy dresses also affect the walking pattern.

#### 5.1.3. Viewpoint Variations

Viewpoint variations are a common vision-based problem. The reason is that any object image’s orientation depends on the camera’s orientation and position. This is also a common problem for gait recognition systems because during the capture of the walking sequences, if the orientation and position of the imaging device are varied for individuals, the captured sequences will be different, which makes it difficult to identify the individuals.

#### 5.1.4. Occlusion and Noise

There are two types of techniques for recognizing gait in defiance of occlusion: reconstruction-free and reconstruction-based methods. Gait Energy Images (GEIs), one feature extracted from gait cycle silhouettes by reconstruction-free methods, offer greater accuracy. These techniques are not applicable to low degrees of occlusion, where it is challenging to determine gait cycles. Reconstruction-based approaches, on the other hand, seek to restore occluded people. These techniques work with numerous gait periods that have some frames partially obscured. However, using this strategy becomes difficult when every frame in a series is highly obscured. The primary disadvantage of reconstruction-based methods is that they frequently make it harder to distinguish between different people because of restored silhouette sequences.

#### 5.1.5. Cross-View Conditions

It is a technique for identifying any person based on their walking pattern. The system can be categorized in three ways. The first way is the three-dimensional representation of gait, which required a different camera to manage. This system is not ideal for public monitoring. The second way only considers the view-invariant gait pattern; however, this process can work only for formal poses, not for other or different poses. In the third way, the person is trained in the transformation model from both viewpoints.

#### 5.1.6. Speed Variations

The speed of the mobility of a person can change the person’s walk. This can change the phase of gait cycle and joint angle movement.

#### 5.1.7. Unconstrained Environment

Gait recognition under unconstrained conditions is still a challenging task. In real-world scenarios, there are various challenges that need to be addressed, such as noise, clutter, different lighting conditions, and occlusions. These challenges make it difficult to obtain accurate and reliable gait features, which may affect the performance of gait recognition systems. Hence, developing gait recognition systems that can handle unconstrained conditions is still an active area of research.

#### 5.1.8. Spatial and Temporal Situations

A Gait Energy Image (GEI) can be used to record information about space (spatial); however, it can be hard to obtain good information about time (temporal), which can make human recognition less accurate. In terms of temporal situations, recognizing gait over long periods of time can be challenging due to the need for continuous and reliable data capture as well as the potential for changes in an individual’s gait pattern over time due to aging or injury. Furthermore, recognizing gait in real-time scenarios requires efficient and faster processing techniques that can operate on the available computing resources in a timely manner. These spatial and temporal challenges highlight the need for further research and development in gait recognition technology to address these limitations and improve its reliability and effectiveness in real-world applications.

#### 5.1.9. Ethical Concerns

The use of gait recognition systems in public spaces raises privacy concerns, as individuals may not want their gait patterns to be recorded or analyzed.

### 5.2. Model-Based Limitations and Challenges

Pose-based gait recognition methods fall into one of two groups of model-based approaches. These models analyze gait patterns using the body’s joint angles; however, they need superior gait segments and a multi-camera system. Since this method includes calculating important points for each frame, it is more expensive than model-free approaches.

#### 5.2.1. Extracting Skeleton Data

Extracting accurate skeleton data from RGB or depth images is still a challenging task, especially in complex scenarios where multiple individuals or occlusions are present. Additionally, due to differences in camera viewpoints and body orientations, the skeleton data may have variations in scale, rotation, and translation.

#### 5.2.2. Interdependency

The gait recognition process based on the skeleton depends on the other methods to estimate the pose. After estimating the pose, the gait recognition method utilized that pose information for gait recognition. So, the complexity of the gait recognition method will increase if the pose estimation process is not working well.

#### 5.2.3. Spatial and Temporal Situations

The representation and modeling of the temporal dynamics of gait patterns from skeleton data are not straightforward, especially when the number of skeleton joints is large. This makes it challenging to capture the spatiotemporal dependencies and dynamics of gait effectively.

#### 5.2.4. Hard Sample Issue

Gait recognition with skeleton data may face identity ambiguity issues where different individuals may have similar gait patterns, which is called the hard sample. Here, the same pedestrian has a distinct skeleton representation and vice versa.

#### 5.2.5. Viewpoints and Positioning

Using skeleton data for gait recognition can be sensitive to changes in the camera’s point of view, which can cause big changes in the skeleton data that were captured. 

#### 5.2.6. Unconstrained Environment

The accuracy of the skeleton-tracking algorithm used to extract the joint positions from the input video data can be affected by factors such as occlusions, a cluttered background, and lighting conditions, leading to missing or erroneous joint positions.

## 6. Problem Identification and Discussion

Deep architecture for gait recognition faces several challenges. One of the challenges is the limited availability of labeled data for training deep networks. Deep networks typically require large amounts of labeled data to avoid overfitting and to generalize well to unseen data. However, gait datasets with labeled data are limited and expensive to acquire, which makes it challenging to train deep networks for gait recognition. Another challenge is the difficulty of designing effective deep network architectures for gait recognition. The architecture should be able to capture both spatial and temporal information effectively and efficiently, which is not trivial. Additionally, designing a deep network architecture that is robust to variations in gait due to changes in clothing, carrying conditions, and other environmental factors is also challenging. Finally, the interpretability of deep networks is also a challenge, as they are often seen as black boxes, making it difficult to understand how they arrive at their decisions.

### 6.1. Problems with Silhouette Images Overcome by Skeleton Structure

For different covariates, silhouette images can lose fine-grained spatial and appearance information in complex scenes. However, specific deep architectures can overcome the specific gait recognition problems. Such skeleton body representation overcomes the problem of silhouette images. The covariate factors of the silhouette image create disparity issues; however, the skeleton data, which are the raw data extracted from the pose estimation algorithms, can overcome these types of limitations.

### 6.2. Problems of Deep Neural Architecture for Processing Skeleton Data

For the feature extraction process, the skeleton data are used in the deep neural architectures. However, the following problems occur during the feature extraction process.

- Deep structures (CNNs) treat the skeleton as grid-shaped structural data, whereas the skeleton is graph-shaped structural data, thus resulting in limited representation and difficulties with generalization.
- Gait patterns are extracted from specific body parts. However, the deep structure lacks the attention mechanisms to emphasize the significant body regions.
- Deep structures (CNNs) are rotationally invariant. For viewpoint changes, we need to be rotationally equivariant.
- Deep structures may struggle to handle gait data captured from different angles and perspectives, which can impact gait accuracy. However, CapsNet can handle this problem.
- As the gait skeleton is composed of a number of non-Euclidean graphs, it is unable to reveal the latent spatial connections in the joints of the skeleton.

Gait recognition is a sequence-based problem, and GNNs are specifically designed to handle structured data. GNNs process data in the form of graphs, where each node represents a feature and edges represent relationships between features. By modeling the gait sequence as a graph, GNNs can capture the relationships between the steps in the sequence and use that information to make gait predictions. GNN allows for the capture of more complex relationships in gait data compared to traditional neural networks, either holistically or partially.

## 7. Conclusions

In recent years, gait recognition has drawn a lot of interest from the research community because that has become a non-invasive and promising method of biometric identification. Deep learning methods have shown great potential in automatically extracting discriminative features for gait recognition. However, recognizing gait accurately is still a challenging task, which is mainly due to the variability and complexity of environments and human body representations. This paper provided a comprehensive overview of the recent advancements in this field, analyzed the performance of state-of-the-art techniques, and presented a taxonomy of deep learning methods used for gait recognition. The limitations and challenges of deep learning in gait recognition were also discussed, and several research directions are suggested to improve the performance of gait recognition. Overall, this paper provides valuable insights into the current state-of-the-art and future research directions in gait recognition using deep learning methods.

## 8. Future Directions

Even though the area of gait recognition has made substantial progress, more study is still required. Although there are now a lot of gait datasets accessible, their use is complicated by their limitations. A lot of data can be produced when taking into account multi-view and multi-angle situations. These datasets, however, are frequently restricted to specific environmental circumstances and are only helpful for single individual detection. As gait recognition technology gains popularity, it has become possible to identify numerous people moving across a crowd in real time. This has given researchers fresh areas to explore. In order to enhance gait recognition, new algorithms must be created that concentrate on the spatiotemporal aspects of movement in a model-free manner. In the future, studies on recognizing gender using gait patterns may also aid in the improvement of gait recognition [7,8,9].

The following list contains a few research directions for future gait recognitions:

- **Multi-modal gait recognition:** In this method, gait recognition can be combined with other types of data, such as facial recognition, voice recognition, or biometric data from wearable sensors. This can help make gait recognition systems more accurate and reliable, especially in tough situations where gait recognition alone might not be enough.
- **Deep learning techniques:** Deep learning models can learn complex features and patterns from large amounts of data, which can potentially improve the accuracy of gait recognition systems. This approach can also help reduce the need for manual feature engineering, which can be time-consuming and challenging.
- **Robustness to environmental factors:** In real-world scenarios, gait recognition systems may encounter various environmental factors such as changes in lighting, weather conditions, and terrain. Developing methods that can handle these variations can improve the accuracy and reliability of gait recognition systems in practical applications.
- **Privacy-preserving gait recognition:** Privacy concerns have been raised regarding the use of full-body images in gait recognition systems. Developing methods that can recognize gait while preserving individual privacy can address these concerns and increase the acceptance and adoption of gait recognition technology.
- **Long-term tracking:** Gait recognition systems that can track individuals over longer periods, such as days or weeks, can provide valuable information for security and surveillance applications. Developing methods that can handle variations in gait due to changes in clothing or footwear can improve the accuracy and reliability of long-term tracking systems.
- **Cross-domain gait recognition:** Gait recognition models trained on one dataset may not generalize well to other datasets with different conditions and populations. Developing methods that can adapt to different datasets can improve the performance and applicability of gait recognition systems across different domains.
- **Real-time gait recognition:** In many real-world scenarios, gait recognition systems need to operate in real time with low computational requirements and fast processing times. Developing real-time gait recognition methods can address these requirements and increase the applicability and adoption of gait recognition technology.

## Figures and Tables

**Table 1:** The summary of the available datasets for gait recognition.

Name of Dataset	Presentation: Subject/Sequences: Environment: Views	Covariates
CASIA-A [66]	RGB: 20/240: Outdoor: 3	Walking in normal
CASIA-B [33]	RGB; Silhouette: 124/13,680: Indoor: 11	Walking: Normal; Carrying—a Bag; Wearing—a Coat
CASIA-C [67]	Infrared; Silhouette: 153/1530: Outdoor: 1	Three Walking Speed; Carrying—a Bag
CASIA-E [61,68]	Silhouette: 1014/Undisclosed: Indoor and Outdoor: 15	Three Scenes; Walk-Normal; Carrying—a Bag; Wearing—a Coat
OU-ISIR [64]	Silhouette: 4007/31,368: Outdoor: 4	Walk-Normal
OU-ISIR LP Bag [65]	Silhouette: 62,528/187,584: Indoor: 1	Carried Objects—7 variations
OU-ISIRMV [62]	Silhouette: 168/4200: Indoor: 25	View—24azimuthviewsandTopview—1
OU-ISIR Speed [69]	Silhouette: 34/306: Indoor: 4	walking speeds—Nine
OU-ISIR Clothing [70]	Silhouette: 68/2746: Indoor: 4	Clothing—up to 32 combinations
OU-MVLP [60]	Silhouette; Skeleton: 10,307/259,013: Indoor: 14	Walk-Normal
OU-MVLP Pose [71]	Skeleton: 10,307/259,013: Indoor: 14	Walk-Normal
TUM GAID [72]	RGB; Depth; Audio: 305/3737: Indoor: 1	Walk-Normal; Backpack; Wearing coat with shoes

**Table 2:** A summary of CNN architectures published in different publications for gait recognition.

Models	Input Dimension	Total Layer	Conv. Layer	Pooling Layer	Fully Connected Layer
PF-Gait [96]	64 × 64	7	3	2	2
Gait-Part [97]	64 × 64	9	6	2	1
GEI-Gait [98]	120 × 120	11	5	4	2
Pose-Gait [99]	64 × 64	6	3	2	1
GaitSET [100]	64 × 64	5	3	2	1
MA-GAIT [31]	124 × 124	8	3	3	2
GEINet [32]	88 × 128	6	2	2	2
Ensem.-CNNs [34]	128 × 128	7	3	2	2
Gait-joint [101]	64 × 64	16	12	2	2
MGANs [102]	64 × 64	8	4	1	3
EV-Gait [103]	128 × 128	9	6	0	2
Gait-Set [37]	64 × 64	9	6	2	1
Caps-Gait [104]	64 × 64	9	6	2	1
SMPL [40]	64 × 64	5	3	1	1
Gait-RNNPart [39]	64 × 64	9	6	2	1

**Table 3:** Deep architectures are presented based on the proposed taxonomy.

Reference	Published Year	Publisher	Venue	Body Shape	Deep Methods	Datasets
[135]	2015	IEEE	IEEE-T-MM	Silhouette	CNN	CASIA-B
[31]	2015	IEEE	IEEE-CISP	Silhouette	CNN	CASIA-B
[15]	2016	IEEE	IEEE-ICPR	Skeleton	LSTM	CASIA-B
[32]	2016	IEEE	IEEE-ICB	Silhouette	CNN	OU-ISIR
[76]	2016	IEEE	IEEE-ICIP	Silhouette	3DCNN	CMU Mobo; USF HumanlD
[136]	2016	IEEE	IEEE-ICASSP	Silhouette	CNN	OU-ISIR
[131]	2016	Journal	BMVC	Skeleton	CNN + LSTM	CASIA-B; CASIA-A
[110]	2017	Inderscience	IndS-Int. J. Biom.	Silhouette	DBN	CASIA-B
[137]	2017	ScienceDir	SD-CVIU	Silhouette	CNN	CASIA-B
[34]	2017	IEEE	IEEE-T-PAMI	Silhouette	CNN	CASIA-B; OU-ISIR
[138]	2017	MDPI	Applied Sci.	Silhouette	CNN	OU-ISIR
[139]	2017	IEEE	IEEE-T-CSVT	Silhouette	CNN	OU-ISIR
[140]	2017	IEEE	IEEE-BIOSIG	Silhouette	CNN	TUM-GAID
[141]	2017	Journal	MM	Silhouette	CNN	OU-ISIR
[142]	2017	IET	IET-CCBR	Skeleton	CNN + LSTM	CASIA-B
[74]	2017	IEEE	IEEE-CVPRW	Silhouette	GAN	CASIA-B
[80]	2017	ScienceDir	SD-NC	Silhouette	DAE	CASIA-B; SZU RGB-D
[122]	2018	Journal	Elect. Imaging	Silhouette	3DCNN	CASIA-B
[83]	2018	IEEE	IEEE-Access	Silhouette	CNN + LSTM	CASIA-C
[117]	2018	SpringerLink	SL-Neuroinform	Silhouette	3DCNN	OU-ISIR
[35]	2018	IEEE	IEEE-DIC	Skeleton	CNN	CASIA-B
[143]	2018	IEEE	IEEE-Access	Silhouette	CNN + LSTM	CASIA-B; OU-ISIR
[123]	2018	IEEE	IEEE-ISBA	Silhouette	3DCNN	CASIA-B
[132]	2018	IEEE	IEEE-ICME	Silhouette	DAE + GAN	CASIA-B
[144]	2018	ScienceDir	SD-JVCIR	Silhouette	CNN	CASIA-B; OU-ISIR
[145]	2018	SpringerLink	SL-CCBR	Skeleton	CNN + LSTM	CASIA-B
[118]	2019	ScienceDir	SD-PRL	Skel.; Silh.	LSTM	CASIA-B; TUM-GAID
[38]	2019	IET	IET-Biom.	Silhouette	CNN	CASIA-B; TUM; OU-ISIR
[36]	2019	IEEE	IEEE-CVPR	Skel.; Silh.	DAE + LSTM	CASIA-B; FVG
[146]	2019	ScienceDir	SD-J. Sys. Arch.	Silhouette	DAE + GAN	CASIA-B; OU-ISIR
[101]	2019	ScienceDir	SD-PR	Silhouette	CNN	CASIA-B; SZU
[102]	2019	IEEE	IEEE-T-IFS	Silhouette	GAN	CASIA-B; OU-ISIR
[147]	2019	ScienceDir	SD-PRL	Silhouette	CNN	CASIA-B; OU-ISIR
[103]	2019	IEEE	IEEE-CVPR	Silhouette	CNN	CASIA-B; OU-ISIR LP Bag
[106]	2019	ScienceDir	SD-NC	Silhouette	GAN	CASIA-B; OU-ISIR
[107]	2019	IEEE	IEEE-IJCNN	Silhouette	GAN	CASIA-B
[125]	2019	IEEE	IEEE-T-IFS	Skeleton	DAE	OU-ISIR LP Bag; TUM-GAID
[148]	2019	Conf.	ICVIP	Silhouette	CNN	CASIA-B
[149]	2019	IEEE	IEEE-T-MM	Silhouette	CNN + LSTM	CASIA-B; OU-ISIR
[108]	2019	IEEE	IEEE-IJCNN	Silhouette	GAN	CASIA-B
[150]	2019	SpringerLink	SL-NCAA	Silhouette	CNN	CASIA-B; CASIA-A; OU-ISIR
[68]	2019	ScienceDir	SD-PR	Silhouette	CNN	CASIA-B
[151]	2019	SpringerLink	SL-NCAA	Silhouette	CNN	CASIA-B; OU-ISIR
[112]	2019	ScienceDir	SD-JVCIR	Silhouette	CapsNet	CASIA-B
[37]	2019	SpringerLink	SL-AAA	Silhouette	CNN	CASIA-B; OU-MVLP
[113]	2019	ScienceDir	SD-JVCIR	Silhouette	CapsNet	CASIA-B; OU-ISIR
[85]	2020	IEEE	IEEE-Access	Skeleton	DAE + LSTM	Walking Gait
[152]	2020	ScienceDir	SD-PR	Skeleton	CNN	CASIA-B; CASIA-E
[133]	2020	IEEE	IEEE-T-PAMI	Silh; Skel	DAE + LSTM	CASIA-B; FVG
[130]	2020	IEEE	IEEE-T-IP	Silhouette	CNN + LSTM	CASIA-B; OU-MVLP; OU-LP
[109]	2020	ScienceDir	SD-PR	Silhouette	GAN	OULP-BAG; OU-ISIR LP Bag
[153]	2020	SpringerLink	SL-MTAP	Silhouette	CNN	CASIA-B
[134]	2020	ScienceDir	SD-KBS	Silhouette	LSTM + Capsule	CASIA-B; OU-MVLP
[154]	2020	Journal	JINS	Silhouette	CNN + LSTM	CASIA-B; OU-ISIR
[155]	2020	IEEE	IEEE-T-CSVT	Silhouette	CNN	CASIA-B; OU-MVLP; OU-ISIR
[124]	2020	arXiv	arXiv	Silhouette	3DCNN	CASIA-B; OU-MVLP
[156]	2020	SpringerLink	SL-MTAP	Silhouette	CNN	CASIA-B; OU-ISIR
[104]	2020	arXiv	arXiv	Skeleton	GCN	CASIA-B
[157]	2020	SpringerLink	SP-MTAP	Silhouette	CNN	CASIA-B; OU-ISIR
[158]	2020	Journal	J-JIPS	Silhouette	CNN	CASIA-B; OU-ISIR
[159]	2020	SpringerLink	SL-MTAP	Silhouette	CNN	CASIA-B
[160]	2020	SpringerLink	SL-SC	Silhouette	CNN	CASIA-B; OU-ISIR; OU-MVLP
[114]	2020	IEEE	IEEE-ITNEC	Silhouette	CapsNet	CASIA-B; OU-ISIR
[40]	2020	IEEE	IEEE-CVPR	Silhouette	CNN	CASIA-B; OU-MVLP
[126]	2020	IEEE	IEEE-CVPR	Silhouette	DAE	CASIA-B; OU-ISIR LP Bag
[71]	2020	IEEE	IEEE-T-Biom	Skeleton	CNN + LSTM	OUMVLP-Pose
[161]	2020	Conf.	C-ACCVW	Silhouette	CNN	CASIA-E
[162]	2020	Conf.	C-ACCVW	Silhouette	CNN	CASIA-E
[115]	2020	IEEE	IEEE-ICPR	Silhouette	CNN + GRU + CapsNet	CASIA-B; OU-MVLP
[39]	2020	IEEE	IEEE-T-Biom.	Silhouette	CNN + GRU	CASIA-B; OU-MVLP
[163]	2020	IEEE	IEEE-Access	Silhouette	CNN	CASIA-B
[164]	2020	IEEE	IEEE-ICASSP	Silhouette	CNN	CASIA-B; OU-MVLP
[165]	2020	IEEE	IEEE-IJCB	Silhouette	DAE + GAN	CASIA-B; OU-ISIR
[42]	2020	CVF	ACCV	Silh; Skel	CNN + LSTM	CASIA-B; OU-MVLP
[41]	2020	ACM	ACM-MM	Silhouette	3DCNN	CASIA-B; OU-ISIR
[43]	2020	SpringerLink	SL-ECCV	Silhouette	CNN	CASIA-B; OU-MVLP
[166]	2020	ScienceDir	SD-NC	Sleleton	CNN	UPCV; KS20; SDU
[167]	2021	SpringerLink	SL-ES	Skeleton	3DCNN	CASIA- B
[44]	2021	SpringerLink	SL-VC	Skeleton	GCNN	CASIA- B
[168]	2021	SpringerLink	SL-ACPR	Skeleton	GAN	CASIA-B; OU-ISIR
[45]	2021	ScienceDir	SD-PR	Skeleton	GCN	TUM Gait
[97]	2021	SpringerLink	SL-JBD	Silhouette	CNN	Market dataset
[169]	2021	SpringerLink	SL-CIS	Image	CNN	CASIA- B
[98]	2021	SpringerLink	SL-SC	Silhouette	CNN	CASIA- B, OU-ISIR, OU-MVLP
[129]	2021	IEEE	IEEE-ICIP	Skeleton	GCNN	CASIA- B
[86]	2021	IEEE	IEEE-T-PAMI	Skeleton	GCN + CNN	CASIA- B
[170]	2021	IEEE	IEEE-PRCV	Skeleton	GCN	OUMVLP-Pose
[46]	2021	IEEE	IEEE-ICCV	Silhouette	3DCNN	CASIA- B; OU-MVLP
[171]	2021	CVF	CVF-CVPR	Silhouette	CNN	OUMVLP
[99]	2021	IEEE	IEEE-ICCV	Skeleton	CNN	CASIA- B; OU-MVLP
[100]	2021	IEEE	IEEE-T-PAMI	Silhouette	CNN	CASIA- B; OU-MVLP
[29]	2021	ScienceDir	SD-ESWA	Silhouette	3DCNN	CASIA- B; OULP
[73]	2021	IEEE	IEEE-T-CSVT	Silhouette	CNN	CASIA- B; OULPOU-MVLP
[172]	2021	ScienceDir	SD-NC	Silhouette	CNN	CASIA- B; OU-MVLP
[88]	2021	IEEE	IEEE-T-BBIS	Silhouette	CNN	CASIA- B; OU-MVLP
[173]	2021	IEEE	IEEE-ICPC	Silhouette	CNN	CASIA- B; OU-MVLP
[47]	2021	IEEE	IEEE-T-IFS	Silhouette	ANN	CASIA-BTUM-GAIT
[96]	2022	ScienceDir	SD-DSP	Silhouette	CNN	CASIA-B, OUMVLP
[174]	2022	CVF	CVF	Silh; Skel	CNN	CASIA-B; OUMVLP
[49]	2022	IEEE	IEEE-Access	Skeleton	GCNN	CASIA-B
[48]	2022	ScienceDir	SD-CVIU	Silh; Skel	GCN + CNN	CASIA-B
[175]	2022	Wiley	Wiley-Expert system	Skeleton	DCNN	CASIA-A; B, C
[89]	2022	ScienceDir	SD-PR	Silhouette	CNN	CASIA-B
[127]	2022	IEEE	IEEE-CVPR	Skeleton	GCN	CASIA-B; OUMVLP-Pose
[128]	2022	ScienceDir	SD-PRL	Skeleton	GCN	CASIA-B; OUMVLP-Pose
[90]	2022	IEEE	IEEE-T-NNLS	Silhouette	CNN	CASIA-B; OUMVLP
[75]	2022	MDPI	Electronics	Skeleton	CNN	CASIA-B
[176]	2022	Taylor	Taylor-CS	Skeleton	GCN	CASIA-B
[50]	2022	MDPI	Sensor	Silhouette	ViT	CASIA-B; OU-ISIR OU-LP
[91]	2022	IEEE	IEEE-T-IP	Silhouette	CNN	CASIA-B; OUMVLP
[92]	2022	ScienceDir	SD-PR	Silhouette	CNN	CASIA-B; OUMVLP

**Table 4:** The performance of the state-of-the-art literature based on CASIA-B.

Information				Performances			
Reference	Year	Publisher	Venue	NM	BG	CL	Avg.
[135]	2015	IEEE	IEEE-T-MM	78.90	-	-	-
[110]	2017	Inderscience	IndS-Int. J. Biom.	90.80	45.90	45.30	60.70
[34]	2017	IEEE	IEEE-T-PAMI	94.10	72.40	54.00	73.50
[35]	2018	IEEE	IEEE-DIC	83.30	-	62.50	-
[68]	2019	ScienceDir	SD-PR	75.00	-	-	-
[102]	2019	IEEE	IEEE-T-IFS	79.80	-	-	-
[101]	2019	ScienceDir	SD-PR	89.90	-	-	-
[36]	2019	IEEE	IEEE-CVPR	93.90	82.60	63.20	79.90
[103]	2019	IEEE	IEEE-CVPR	89.90	-	-	-
[38]	2019	IET	IET-Biom.	94.50	78.60	51.60	74.90
[118]	2019	ScienceDir	SD-PRL	86.10	-	-	-
[37]	2019	SpringerLink	SL-AAA	**95.00**	**87.20**	**70.40**	**84.20**
[133]	2020	IEEE	IEEE-T-PAMI	92.30	88.90	62.30	81.20
[130]	2020	IEEE	IEEE-T-IP	96.00	-	-	-
[155]	2020	IEEE	IEEE-T-CSVT	92.70	-	-	-
[163]	2020	IEEE	IEEE-Access	95.10	87.90	74.00	85.70
[115]	2020	IEEE	IEEE-ICPR	95.70	90.70	72.40	86.30
[39]	2020	IEEE	IEEE-T-Biom.	95.20	89.70	74.70	86.50
[40]	2020	IEEE	IEEE-CVPR	96.20	91.50	78.70	88.80
[126]	2020	IEEE	IEEE-CVPR	94.50	-	-	-
[43]	2020	SpringerLink	SL-ECCV	96.80	94.00	77.50	89.40
[42]	2020	CVF	ACCV	**97.90**	**93.10**	77.60	89.50
[41]	2020	ACM	ACM-MM	96.70	93.00	**81.50**	**90.40**
[44]	2021	SpringerLink	SL-VC	97.03	90.77	89.90	92.57
[46]	2021	IEEE	IEEE-ICCV	98.30	**95.50**	84.50	92.77
[47]	2021	IEEE	IEEE-T-IFS	97.70	94.80	95.30	95.93
[100]	2021	IEEE	IEEE-T-PAMI	96.10	90.80	70.30	96.10
[173]	2021	IEEE	IEEE-ICPC	96.20	92.90	87.20	92.10
[29]	2021	ScienceDir	SD-ESWA	-	-	-	98.34
[45]	2021	ScienceDir	SD-PR	**99.40**	95.40	**99.40**	**98.07**
[49]	2022	IEEE	IEEE-Access	-	-	-	98.86
[48]	2022	ScienceDir	SD-CVIU	**97.70**	93.80	92.70	94.73
[75]	2022	MDPI	Electronics	94.00	**95.00**	**97.00**	95.33
[50]	2022	MDPI	Sensor	-	-	-	**99.93**
[91]	2022	IEEE	IEEE-T-IP	97.50	94.50	88.00	93.33
[92]	2022	ScienceDir	SD-PR	96.70	92.40	81.60	90.23

NM = Normal walk; BG = Carrying bag; CL = Walk with wearing coat.

**Table 5:** The performance of the state-of-the-art literature based on OU-MVLP.

Information				
Reference	Published Year	Publisher	Venue	Performances
[37]	2019	SpringerLink	SL-AAA	83.40
[164]	2019	IEEE	IEEE-ICASSP	57.80
[155]	2020	IEEE	IEEE-T-CSVT	63.10
[130]	2020	IEEE	IEEE-T-IP	84.60
[115]	2020	IEEE	IEEE-ICPR	84.50
[39]	2020	IEEE	IEEE-T-Biom.	84.30
[40]	2020	IEEE	IEEE-CVPR	88.70
[43]	2020	SpringerLink	SL-ECCV	89.18
[46]	2021	IEEE	IEEE-ICCV	90.90
[100]	2021	IEEE	IEEE-T-PAMI	87.90
[73]	2021	IEEE	IEEE-T-CSVT	94.92
[88]	2021	IEEE	IEEE-T-BBIS	96.40
[173]	2021	IEEE	IEEE-ICPC	89.90
[98]	2021	SpringerLink	SL-SC	98.00
[90]	2022	IEEE	IEEE-T-NNLS	96.15
[91]	2022	IEEE	IEEE-T-IP	90.50
[92]	2022	ScienceDir	SD-PR	89.30

## Data Availability

Not applicable.
